# Metal Exposures in Residents Living Near an Urban Oil Drilling Site in Los Angeles, California

**DOI:** 10.1021/acs.est.2c04926

**Published:** 2022-10-26

**Authors:** Arbor J. L. Quist, Yoshira Ornelas Van Horne, Shohreh F. Farzan, Jill E. Johnston

**Affiliations:** Department of Population and Public Health Sciences, Keck School of Medicine, University of Southern California, 1845 N Soto St, Los Angeles, California90032, United States

**Keywords:** metal mixtures, environmental justice, toenail, biomarker, exposure

## Abstract

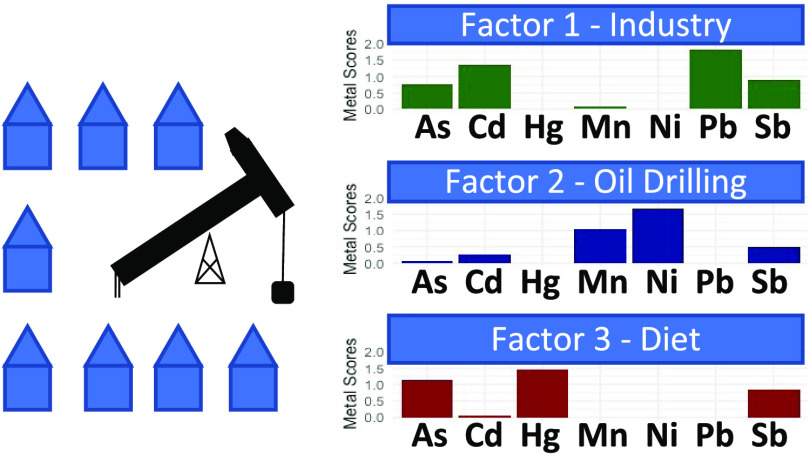

Urban environmental justice communities are potentially exposed to multiple toxic metals, through contaminated air, soil, water, and food. However, information on metals and their sources is lacking. This study uses non-negative matrix factorization (NMF) in a community-based participatory research study to identify potential sources and to understand how these metals cluster in a population near an urban oil drilling site. We recruited 203 Latinx, Black, and Asian residents who lived within 1 km of an oil drilling site in south Los Angeles and collected toenail clippings to assess exposure to arsenic (As), cadmium (Cd), mercury (Hg), manganese (Mn), nickel (Ni), lead (Pb), and antimony (Sb). Using NMF, we identified three clusters based on concentrations in the participants’ toenails. As, Cd, Pb, and Sb grouped together, indicative of an industrial source. A second grouping was composed of Ni and Mn, which may be related to oil drilling. We also identified a third source factor predominantly driven by Hg and As, which may arise from dietary sources. Utilizing NMF, a dimension reduction method, we identified a source factor high in Ni and Mn in residents living in a neighborhood near an active oil drilling site.

## Abstract 2

Mixture methods may be useful in understanding the sources of contaminants in areas with multiple exposures. This study used a mixture method to identify a source factor high in nickel and manganese, which may be related to a nearby active oil drilling site.

## Introduction

Urban communities are regularly exposed to various toxic metals from nearby industries, traffic, and contaminated water, soil, or food.^1,2^ Los Angeles (LA) County, California, is the largest urban oil field in the United States with over 5000 active wells among a population of nearly 10 million people. Land development, population growth, and oil exploration in Los Angeles occurred concurrently, leaving a patchwork of thousands of active oil wells operating in very close proximity to homes, schools, and parks.^3^ Few protections are in place to prevent the release of pollutants into nearby residential areas.^3^ Urban oil drilling often exposes the surrounding communities to multiple hazardous pollutants, including toxic metals. Chemicals associated with oil extraction and production include carcinogens, mutagens, reproductive toxins, developmental toxins, and endocrine disruptors.^4,5^

Recent research demonstrates multiple health-hazardous pollutants associated with petroleum extraction, including toxic metals.^6^ Crude oil contains metals such as cadmium (Cd), lead (Pb), manganese (Mn), nickel (Ni), and vanadium (V), and drilling fluids may additionally contain additional metals like chromium (Cr) and zinc (Zn).^7,8^ Drinking water near oil fields in Bolivia has been found to contain aluminum (Al), arsenic (As), iron (Fe), and Mn.^9^ Soils near oil extraction operations and drilling wastes have been found to contain Cd, copper (Cu), Cr, Mn, Pb and Zn at significantly higher levels compared to background concentrations.^10−12^ The concentrations of Ni, Mn, and Cd in soil have been found to increase significantly with the number of years the well has been producing, with the highest concentrations found near the oldest active oil wells (producing > 40 years).^10^ Additionally, a study in Southwestern Los Angeles County found oil field operations to be associated with increases in airborne Mn and Ni concentrations.^13^

While petroleum extraction is increasingly common in urbanized areas and has been occurring for over a century without a systematic understanding of exposure risks, remarkably little is known about the exposure for nearby residents.^5^ There are no buffer requirements in LA for existing wells, so homes, schools, and parks often sit within meters of the drilling operations. In this study, we partnered with a community near along the Las Cienagas oil fields in South Los Angeles. According to the 2010 US Census, over 90% of residents are people of color (self-identify as Latinx and/or as a race other than White). CalEnviroScreen, the state of CA’s environmental justice screening tool that uses environmental, health, and socioeconomic data to identify highly burdened and vulnerable communities, revealed that this area is among the top 15% most environmentally burdened communities in the state.^14^ Proximity to oil and gas development is not considered in CalEnviroScreen; many communities near drill sites face multiple environmental burdens and social vulnerabilities. Understanding the impacts of drilling operations on the health and welfare of this community is critical to improving the public health conditions for this neighborhood (as well as in other similar communities). To advance our understanding of potential metal exposure in residents near urban oil drilling sites, we analyzed metal concentrations in toenail samples of residents living within 1 km of the drilling site. To better characterize potential sources and patterns of exposure in this environmental justice community, we leveraged unsupervised dimension reduction techniques.

## Methods

### Study Population and Area

For this cross-sectional, community-based study, we recruited 239 residents who lived within 1 km of an active oil extraction site in the Las Cienegas oil field in South Los Angeles, CA. The oil extraction site consists of 28 wells, is situated in the Jefferson Park neighborhood, and began operations in the 1960s. Esperanza Community Housing, a community-based organization, worked with our academic research team to train Promotores de Salud (community health workers with connections to the neighborhood) who supported the research as part of the larger Health and Air Pollution Study.^15^ Eligible participants lived within 1 km of the oil drilling site for at least 2 years, were at least 6 years old, and spoke English, Spanish, or Korean. All participants aged 18 years and older gave written informed consent. Assent and parental consent were obtained from participants under age 18. All participants completed a health questionnaire in their preferred language. A parent or guardian completed the questionnaire when the participant was younger than 13 years.

### Toenail Collection and Metal Assessment

We collected toenail clippings to measure exposure to toxic metals because they are noninvasively collected, easily stored, and reflect metals exposure approximately 3–12 months prior to collection.^16^ Toenails were collected from January 2017 to August 2019, typically when participants came to the community site for the study, although 14 toenail kits were taken home and returned within 1–2 weeks. Toenail clippings were cut from all ten toes, and any nail polish was removed beforehand. The mean mass of the toenail clippings was 0.050 ± 0.047 g (range: 0.002–0.286).

The Dartmouth Trace Element Core Facility analyzed the toenail samples for a panel of metals that included arsenic (As), cadmium (Cd), mercury (Hg), manganese (Mn), nickel (Ni), lead (Pb), and antimony (Sb). The nail clippings were first cleaned and washed 5x in an ultrasonic bath using Triton X-100 and acetone. Next, the nails were sonicated with deionized water, freeze-dried, weighted, and digested in Optima HNO_3_ by low-pressure microwave digestion.^17^ The samples were then analyzed for each element μg total per gram (μg/g) by inductively coupled plasma-mass spectrometry on an Agilent 7700X (Agilent Technologies Headquarters, Santa Clara, California). Each analysis included a duplicate analysis of digested toenail samples, spikes of digested samples, and blank and fortified blank digests as quality control measures. Recovery criteria ranged from 80 to 120% of the spike amount for all analytes. Dartmouth Trace Element Core Facility participates in a proficiency-testing program (QEMGAS, Center for Toxicology, Quebec, Canada) and follows quality control procedures found in EPA SW 846 and ETA method 6020 (Test Methods for Evaluating Solid Waste, Physical/Chemical Methods, EPA publication SW-846, Third Edition, Final Updates I (1993), II (1995), IIA (1994), IIB (1995), III (1997), IIIA (1999), IIIB (2005), IV (2008), and V (2015)).^18,19^ Pb concentrations above the level of detection were detected in every participant. Toenail levels of Mn, As, Cd, Sb, Ni, and Hg were detected in 97.7, 99.0, 95.5, 99.1, 99.1, and 93.7% of participants, respectively. We used the level of quantification when the sample was below the level of detection and assigned the limit of detection (approximated at 0.005) divided by the square root of two to samples with no estimated concentration.

### Statistical Methods

We excluded outliers first by removing samples above the mean concentration plus 3 times the standard deviation for each metal. Using this method, we excluded 19 participants because at least one of their toenail metal levels were classified as an outlier (1 Mn, 3 As, 3 Cd, 3 Sb, 6 Pb, 3 Ni). We excluded 17 additional participants because of Ni concentrations above 40 μg/g, possibly due to nail clipper contamination. Original Ni concentrations ranged from 0.05 to 2762.21, and 40 μg/g was chosen as a very high, but reasonable cut point based on the literature.^20^ We examined the distributions of the individual metals and calculated median, interquartile range, minimum, maximum, percentiles, and percent detected for each metal. Because age can be associated with toenail metal concentration, we ran age-adjusted models and used the residuals from these models for non-negative matrix factorization (NMF). We used NMF to understand how the metals group together in an attempt to identify sources in a population near an urban oil drilling site, as this technique has been used previously to understand source signatures using biological samples.^21^ NMF is an unsupervised reduction technique similar to principal component analysis (PCA), but NMF requires matrices to be non-negative and does not impose an orthogonality constraint. Negative exposure values are difficult to comprehend and do not easily indicate the absence of an exposure. NMF finds an approximation of *X* ≈ *WH*, where *X* is a matrix of data with *n* rows (the measured features, in our case, metal concentrations) and *p* columns (samples of study participants), *W* is an *n* × *r* matrix, and *H* is an *r* × *p* matrix. The factorization rank, *r*, or number of groups/sources into which the data are reduced, is a non-negative integer that is much smaller than *n* and *p*. All matrices are non-negative, enabling intuitive interpretation. NMF estimates W and H by finding a local minimum of the following loss function (*D*)
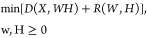
where *R* is an optional regularization function to coerce appropriate smoothness and sparsity on *W* and *H*. We used the R package NMF in R Version 4.1.0, and we tested factorization ranks from 2 to 6.^22^

We also examined the Spearman correlations between the individual metals and between the metals and the source factors. We split the source factors and individual metals into tertiles and examined the characteristics of the participants in each factor. Because we observed a large portion of children in the high group for two of the factors, we also conducted an NMF sensitivity analysis that excluded all children under age 18. Additionally, we examined the characteristics of participants with low, medium, and high concentrations of each individual metal in Supporting Information tables. We looked at differences in source factors by environmental (upwind/downwind of oil drilling, distance to highway), demographic (age, sex), and housing variables (apartment, home). This information was collected from participants through a survey, and we calculated distance to highway and oil drill site using participants’ home address.

## Results

We obtained toenail samples from 239 participants. After excluding participants with any outliers, we were left with 203 participants for the subsequent analyses. About 20% of participants (*n* = 39) were under 18 years of age, while almost 40% were >60 years old (*n* = 75). Over 60% of participants were Hispanic/Latinx, with 22% identifying as Black, 9% as Asian (specifically Korean), and 12% as multiracial. Only 7% of participants were current smokers and 38% were male. All participants lived within 1 km of the oil drilling site, with minimum distance 113 m and maximum distance 970 m.

The median concentrations of As, Cd, Hg, and Sb were 0.07, 0.01, 0.05, and 0.04 μg/g, respectively (Table 1). The median values of Mn (0.26 μg/g) and Pb (0.41 μg/g) were slightly higher, with the Ni median value being the highest (4.69 μg/g). The maximum values were also the highest for Mn (8.24 μg/g) and Ni (38.40 μg/g), after excluding outliers. We observed the strongest correlations between Cd and Pb (ρ = 0.73) and Pb and Mn (ρ = 0.64, Table 2). Ni did not strongly correlate with any of the metals and was only weakly correlated with Mn (ρ = 0.23).

**Table 1:** Metal Concentrations in Toenails (μg/g) of the 203 Participants^a^

				percentile						
metal	detect	median	IQR	min	5%	25%	50%	75%	95%	max
As	99.1	0.07	0.05	0.07	0.03	0.05	0.07	0.10	0.17	0.34
Cd	95.5	0.01	0.01	0.01	0.001	0.004	0.01	0.01	0.05	0.11
Hg	93.7	0.05	0.07	0.05	0.004	0.03	0.05	0.09	0.24	0.52
Mn	97.7	0.26	0.35	0.26	0.07	0.17	0.26	0.51	1.28	8.24
Ni^b^	99.0	4.69	10.93	4.43	0.13	1.21	4.43	12.19	31.49	38.40
Pb	100	0.24	0.41	0.24	0.03	0.11	0.24	0.52	1.88	4.59
Sb	99.1	0.04	0.06	0.03	0.01	0.02	0.03	0.08	0.16	0.27

a For all metals, outliers above 3 times the standard deviation plus the mean for each metal were removed and are not included in this table.

b 20 Ni outliers > 40 μg/g were removed from analysis and this table.

**Table 2:**
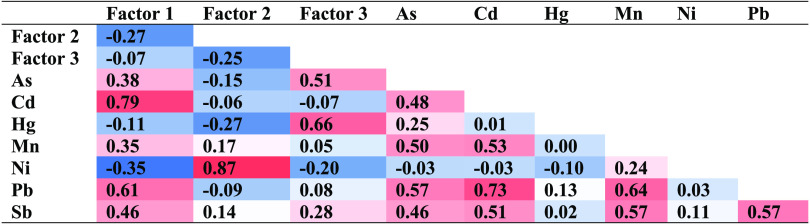
Spearman Correlations between Individual Metals in Toenails and Source Factors

Participants with high As, Cd, Mn, Pb, and Sb concentrations were younger than those with lower levels of each of these metals (Supporting Information, Tables S1, S2, S4, S6, and S7). Participants with the highest tertile of As concentration were more likely to identify as Asian and live closer to the highway. Asian participants were also over-represented in the highest tertile of Hg concentration. Participants with high Hg concentrations tended to live downwind of and near the oil drilling site, as well as near the highway (Supporting Information, Table S3). Participants with a high concentration of Mn were more likely to be Hispanic, never smokers, live farther from the drilling site, and were less likely to live in an apartment building (Supporting Information, Table S4).

While female participants were more likely to be in the highest and lowest tertile of Ni concentration, the Ni concentration distributions of male and female participants were relatively similar (Table S5 and Figure S1). Participants in the highest tertile of Ni concentration were more likely to live 200–1000 m upwind from the drilling site and in houses instead of apartment buildings. Participants with high Pb levels tended to live farther, on average, from the drilling site than those in the lowest tertile of Pb concentration (Supporting Information, Table S6). Participants with high Sb concentrations were more likely to be Hispanic, to not currently work outside the home (predominantly retired), and to live 200–1000 m downwind from the drilling site (Supporting Information, Table S7).

**Figure 1:**
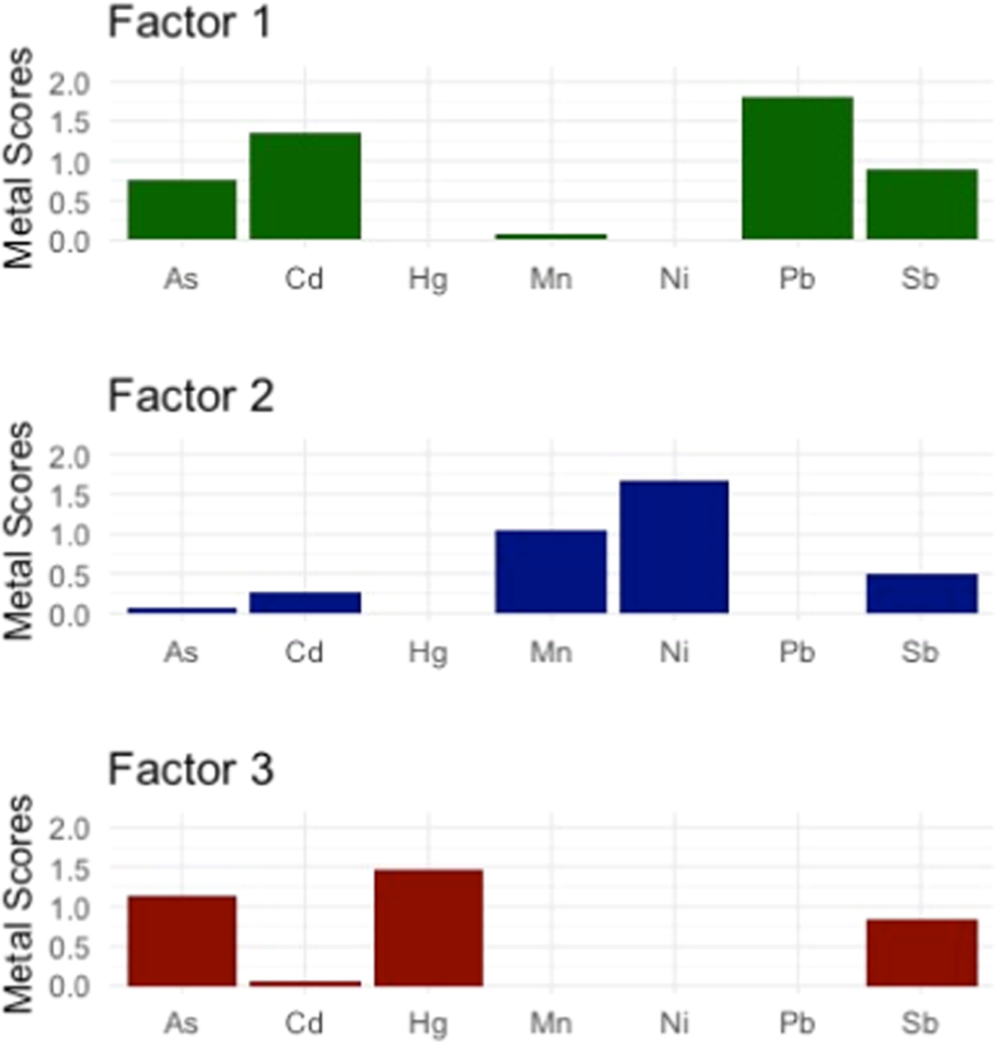
NMF analysis results indicating the three source factors (*n* = 203 participants).

For NMF, we looked at groupings of two to six factors and chose three as the best value based on the cophenetic correlation coefficient, factor sparseness, and residual sum of squares values. In the first factor, Pb, Cd, Sb, and As were grouped together, possibly associated with industrial and traffic-related exposures (Figure 1).^21,23−25^ The second factor was predominantly composed of Ni and Mn. We hypothesize this factor is a marker related to the nearby oil drilling site, as higher levels of Ni and Mn have been observed near oil drilling sites in Los Angeles.^13^ The third factor consists of Hg, As, and Sb, which may be related to dietary sources.^26,27^ Sb was present in all three factors.

We found the industrial source factor (factor 1) to be strongly correlated with age, with higher concentrations in younger children (Table 3). The oil drilling factor (factor 2) was weakly correlated with race; Black participants were more likely to have medium to high levels of factor 2 while Asian residents were more likely to have low levels. The majority of people in the upper tertile of the oil factor live in houses (not in apartments) west of the drilling site. The dietary factor (factor 3) was slightly correlated with distance/direction to drilling site, with a larger percent of participants in the highest tertile living downwind and <200 m from the drilling site. However, we also observed that Asian participants tended to have higher levels of dietary factor and also lived closer to the drill site, on average.

**Table 3:** Participant Characteristics by Tertile of NMF Source Factor, *n* (%), with *p-*Value across Grouping

	factor 1 (Pb, Cd, Sb, As)				factor 2 (Ni, Mn)				factor 3 (Hg, As)			
characteristics	low	medium	high	*p*	low	medium	high	*p*	low	medium	high	*p*
	66	67	67		66	67	67		66	67	67	
age												
<18	10 (15.2)	8 (11.9)	21 (31.3)	0.002	15 (22.7)	15 (22.4)	9 (13.4)	0.7	8 (12.1)	13 (19.4)	18 (26.9)	0.03
18–39	11 (16.7)	12 (17.9)	5 (7.5)		10 (15.2)	7 (10.4)	11 (16.4)		8 (12.1)	14 (20.9)	6 (9.0)	
40–59	28 (42.4)	16 (23.9)	14 (20.9)		15 (22.7)	20 (29.9)	22 (32.8)		28 (42.4)	14 (20.9)	16 (23.9)	
≥60	17 (25.8)	31 (46.3)	27 (40.3)		26 (39.4)	25 (37.3)	25 (37.3)		22 (33.3)	26 (38.8)	27 (40.3)	
sex												
female	38 (57.6)	44 (65.7)	42 (62.7)	0.6	40 (60.6)	39 (58.2)	44 (65.7)	0.7	46 (69.7)	43 (64.2)	35 (52.2)	0.1
male	28 (42.4)	23 (34.3)	25 (37.3)		26 (39.4)	28 (41.8)	23 (34.3)		20 (30.3)	24 (35.8)	32 (47.8)	
race/ethnicity												
Asian	6 (9.1)	8 (11.9)	4 (6.0)	0.7	8 (12.1)	7 (10.4)	3 (4.5)	0.6	1 (1.5)	1 (1.5)	16 (23.9)	<0.001
Black/African American	15 (22.7)	12 (17.9)	16 (23.9)		11 (16.7)	14 (20.9)	18 (26.9)		12 (18.2)	17 (25.4)	14 (20.9)	
hispanic/latinx	40 (60.6)	45 (67.2)	43 (64.2)		44 (66.7)	43 (64.2)	41 (61.2)		49 (74.2)	47 (70.1)	32 (47.8)	
multiracial	5 (7.6)	2 (3.0)	4 (6.0)		3 (4.5)	3 (4.5)	5 (7.5)		4 (6.1)	2 (3.0)	5 (7.5)	
recent smoker												
no	63 (95.5)	62 (92.5)	62 (92.5)	0.7	65 (98.5)	60 (89.6)	62 (92.5)	0.1	63 (95.5)	61 (91.0)	63 (94.0)	0.6
yes	3 (4.5)	5 (7.5)	5 (7.5)		1 (1.5)	7 (10.4)	5 (7.5)		3 (4.5)	6 (9.0)	4 (6.0)	
ever cigarettes												
no	41 (62.1)	47 (71.2)	50 (74.6)	0.3	51 (77.3)	43 (64.2)	44 (66.7)	0.2	45 (68.2)	46 (69.7)	47 (70.1)	0.97
yes	25 (37.9)	19 (28.8)	17 (25.4)		15 (22.7)	24 (35.8)	22 (33.3)		21 (31.8)	20 (30.3)	20 (29.9)	
distance to well												
<250 m	28 (42.4)	27 (40.3)	23 (34.3)	0.6	31 (47.0)	27 (40.3)	21 (31.3)		24 (36.4)	23 (34.3)	32 (47.8)	0.2
250–1000 m	38 (57.6)	40 (59.7)	44 (65.7)		35 (53.0)	40 (59.7)	46 (68.7)	0.2	42 (63.6)	44 (65.7)	35 (52.2)	
direction of well												
east	30 (45.5)	46 (68.7)	45 (67.2)	0.009	46 (69.7)	41 (61.2)	33 (49.3)	0.1	37 (56.1)	45 (67.2)	38 (56.7)	0.3
west	36 (54.5)	21 (31.3)	22 (32.8)		20 (30.3)	26 (38.8)	34 (50.7)		29 (43.9)	22 (32.8)	29 (43.3)	
distance of highway												
<500 m	30 (45.5)	33 (49.3)	38 (56.7)	0.4	36 (54.5)	31 (46.3)	34 (50.7)	0.6	26 (39.4)	31 (46.3)	43 (64.2)	0.01
≥500 m	36 (54.5)	34 (50.7)	29 (43.3)		30 (45.5)	36 (53.7)	33 (49.3)		40 (60.6)	36 (53.7)	24 (35.8)	
apartment												
no	38 (57.6)	35 (52.2)	31 (46.3)	0.4	29 (43.9)	31 (46.3)	45 (67.2)	0.01	38 (57.6)	33 (49.3)	34 (50.7)	0.6
yes	28 (42.4)	32 (47.8)	36 (53.7)		37 (56.1)	36 (53.7)	22 (32.8)		28 (42.4)	34 (50.7)	33 (49.3)	
above 3rd floor												
no	59 (89.4)	53 (79.1)	55 (82.1)	0.3	52 (78.8)	56 (83.6)	59 (88.1)	0.4	60 (90.9)	59 (88.1)	49 (73.1)	0.01
yes	7 (10.6)	14 (20.9)	12 (17.9)		14 (21.2)	11 (16.4)	8 (11.9)		6 (9.1)	8 (11.9)	18 (26.9)	

As some of the highest concentrations of As, Cd, Mn, Pb, and Sb were observed in children, and similarly many participants in the highest tertile of the industrial factor were children (Table 3), we also conducted NMF analysis stratifying by adults and children. These factors were very similar when restricted to participants under age 18 years, indicating that our age adjustment may be adequate (Supporting Information, Figures S2 and S3). Because some participants lived in the same households, we conducted a sensitivity analysis where we added a fixed effect for household while age-adjusting the metal concentrations (Supporting Information, Figure S4); these results were very similar to our main results.

## Discussion

Using NMF, we found that the toenail metal concentration data grouped into three factors, which are consistent with known exposures related to industrial sources, oil drilling, and dietary exposures, which may represent three main sources in this urban environmental justice community. Factor 1, consisting of Pb, Cd, Sb, and As, may represent traffic or industrial pollution from historical sources that continues to contaminate the soil and dust.^21,23−25^ We observed higher levels of the industrial factor among children. Factor 2 consists predominantly of Mn and Ni and may be related to the nearby active oil drilling site.^13^ Factor 3 consists of Hg As, and Sb—which may be from diet—and Asian participants tended to have the highest levels.^26,27^

Sb, Cd, and Pb have been linked to traffic, which may explain why participants in the highest tertile of the industrial factor (the highest Sb of all groups) live closer to the highway on average compared to those in the lowest tertile of the industrial factor (see Supporting Information, Figure S5, map of oil drill site, nearby highway, and approximate locations of participant homes). Studies have found Pb and Cd concentrations in soil to be inversely correlated with distance to road.^28,29^ Sb is released during the breaking process of cars.^30^ As and Sb have similar chemistry and binding properties, which may explain why they grouped together despite typically having different sources.^30^ Pb and Cd are also frequently found together, especially in household dust.^31,32^ Legacy Pb contamination is often found in soil and dust near roadways because of the historical use of leaded gasoline.^25^ Several studies have found higher concentrations of As, Cd, and Pb in urban areas with a higher percent of racial and ethnic minorities compared to whiter urban areas.^24,33^ Additionally, another Los Angeles study of children’s toenails in a different industrial corridor also grouped Sb, Pb, As, and Cd together using NMF.^21^ In our study, the majority of child participants had exposures in the highest tertile of the industrial factor, which may be due to children playing on the ground and in soil or due to differences in these metals accumulating in small bodies. While study results have been inconsistent on the association of metal toenail concentration with age,^16,34^ age can affect toenail metal concentrations as the total amount and proportion of metals in the body and toenail levels are often related to age, as well as toenail rate of growth.^16,35^

Factor 2 is primarily composed of Mn and Ni, which may be indicative of an oil drilling source factor. Ni is the most abundant trace metal in oil and Mn has been linked to oil drilling; both Mn and Ni have been found together near drilling sites in the Los Angeles area.^6,13,36^ Although Mn concentrations in crude oil are typically low, high Mn levels have been seen in California oils.^37,38^ An air quality study in the Inglewood Oil Field and about 2 km from our study site used an X-ray fluorescence spectrometer to measure airborne metals in particulate matter in the fall and winter of 2012–2013. They found that the oil field operations were associated with increases in Mn and Ni concentrations.^13^ Their study used positive matrix factorization (a method similar to NMF) to determine factors that grouped together. Their Mn and Ni factor was associated with winds from the oil field and was low during the holidays (when oil operations are typically reduced). Their results support our hypothesis that the Mn- and Ni-dominated factor is related to the nearby drill site. While Mn and Ni can sometimes enter the body through diet and drinking water, these two elements are not typically found in high levels in the same foods (thus are unlikely to cluster together in a single factor) and Los Angeles municipal water does not report elevated levels of Mn or Ni.^39−41^

Our oil factor also contains some Cd; Cd and Mn have been associated with oil drilling and oil spills.^6,42,43^ In a study of 29 pregnant women living in hydraulic fracturing, median concentrations of urinary and hair Mn in participants were higher than in the general Canadian population.^6^ We did not observe a significant spatial gradient for the oil factor, suggesting that residents across the area may be impacted by the pollution source and that there may not be much of an exposure gradient within 1 km. Epidemiological studies have shown health effects for residents living within 1 km of oil and drilling sites, and several studies indicate increased air pollution and reduced health within 3 km.^44−50^ We observed the Mn and Ni factor to be highest in people who live in homes instead of apartments, which may have to do with exposure to soil. People in homes may have more soil exposure than people in apartments, and soil may be one of the main sources of metal exposure. In a study near an abandoned mining area, Mn dust concentrations were significantly higher in single-family homes than in apartments and mobile homes.^51^

While biomarker studies related to oil drilling are limited, toxic metals in hair, urine, or blood have been used as an indicator of exposure. For example, oil extraction is a known source of elemental mercury (Hg) and higher levels have been identified in hair of people living near oil extraction regions in the Amazon^52^ and in the indoor air in New Mexico in houses above an oil waste pit compared to populations living farther away.^53^ Animal studies have seen higher levels of Pb and Cd in the organs of livestock raised near oil wells.^54^ A study among workers at an oil spill cleanup site in Spain suggested that exposure to the oil mixture presented significant increases in blood concentrations of Al, Ni, and Pb compared to controls; and exposed workers had more endocrine alternations.^55,56^ A pilot study among pregnant women living in natural gas extraction region in rural Canada found elevated concentrations of Al, Mn, barium (Ba), and strontium (Sr) in the hair of the study population compared to the reference population.^6^

The dietary factor that we identified consists predominantly of Hg and As, metals that are commonly found in food and water, and especially in rice and seafood.^26,27^ Studies with the highest toenail As concentrations have largely been in Asian countries, especially Bangladesh, China, and India; this is likely due to arsenic-contaminated water.^34^ We also observed higher As levels among Asian participants, which may be due to a diet high in rice.^57^ Some studies have found consumption of rice, cereal, fish, and seafood to be associated with increased As toenail levels.^34,58^ Additionally, elevated Hg levels in toenails have been consistently positively associated with fish intake.^59^ While our study did not include a food frequency questionnaire, Asian Americans typically eat more fish and rice than Black, Hispanic, or White Americans.^60^ The arsenic measured in this study is predominantly inorganic arsenic, as inorganic arsenic has a high affinity for sulfhydryl groups and thus accumulates in sulfhydryl-rich keratin tissues, including nails.^34^ The small amounts of Cd and Sb in this third factor may also be from diet; diets with leafy greens, tofu, organ meats, and eggs are associated with urinary Cd.^61,62^ One study found a vegetarian diet to be associated with lower concentrations of Sb and Cd.^63^ While we observed higher levels of the dietary factor among residents living downwind and <200 m from the drilling site, this may be because most of the Asian participants lived in the same apartment complex near the drilling site.

Overall, the average toenail concentrations measured in this study were lower than those from other studies of highly polluted communities. Many studies have examined communities near coal ash facilities, industrial corridors, smelters, and other industrial metal polluters. Compared to a study of toenails from 20 children in a smelting village in Vietnam (means: As: 0.36 μg/g, Cd: 0.29, Pb: 157, Mn: 7.41, Hg: 2.63), the metals in the toenails of our participants were relatively low.^64^ The Vietnam study, as well as other studies, observed positive correlations between Mn and Cr, Pb and Cd, Mn and Cd, As and Cd, and As and Mn;^34^ we similarly observed positive correlations between Mn and Cr, Pb and Cd, and As and Mn. Mean Mn levels in toenails are typically below 10 μg/g^16^—as we observed—although higher Mn concentrations in toenails have been found in people living near industrial cities and in other highly polluted areas, as well as in welders.^16^ A study of 95 children living near an industrial corridor (approximately 35 km from the location of this study’s participants) found similar toenail metal concentrations to that of the children in this study (Supporting Information, Table S8), with metal concentrations slightly lower in this study’s cohort aside from Cd.^21^ That study also used NMF to identify source factors, including a dietary source factor (Se, Hg), an industrial source factor (Sb, Pb, As, Cd), and a Mn source factor.

Although the toenail metal concentrations in this study are generally lower than those in other studies of residents in heavily polluted areas, many researchers as well as the US Environmental Protection Agency and Centers for Disease Control and Prevention have shown that there is no safe level of Pb exposure.^65^ Additionally, even small levels of As and Cd exposure can be harmful to human health.^66^ High toenail As levels have been associated with an increased risk of bladder and lung cancer as well as adverse cardiovascular and respiratory outcomes.^34,67^ Studies have also found an association of elevated As with cardiovascular disease mortality in adults, gestational diabetes mellitus in adults, and diarrhea and respiratory symptoms in infants.^68−70^ Elevated Mn exposure is associated with neurological illnesses like Parkinson’s disease and Ni exposure has been associated with lung cancer and cardiovascular diseases.^41,71^ High Ni levels have also been linked to type 2 diabetes.^72,73^

This study is limited by a relatively small sample size and its lack of a comparison group. In this study, we only collected toenails from participants who live <1 km from the oil drilling site, so we are unable to compare high- and low-exposed participants. Participants who live 1 km from an oil drilling site are likely still exposed to chemicals and metals from the drilling. Additionally, there are multiple other active and inactive oil drilling sites within 3 km of this study site, so we are unable to adequately assess variations in exposure, as participants living farther and/or upwind from this drill site might live a mile downwind from a different drill site. A study by Zierold et al. examined residents who lived within 10 miles (16 km—much larger than our study area) from a coal ash storage facility and used geospatial analysis to confirm that the participants with high iron (Fe), Al, and silicon (Si) (which loaded together in nails of children near coal ash storage facility, using PCA) lived closer to the coal ash storage facility.^74^ A study of 39 children in Chicago observed higher toenail levels of Cd, Co, Fe, Mn, and V in children living near an industrial corridor than in children in a comparison community.^75^ Future studies can build on this current study by examining metal concentrations in people near an oil drilling site and using a larger study radius or a control area.

A strength of this study is its use of NMF, which reduces the dimension of our exposure data without using negative values, increasing interpretability. Additionally, we use toenail samples to objectively measure metal concentrations. As with most human biomonitoring studies, biomonitoring is dependent on the sensitivity of the instruments and methods used, and it can be difficult to determine the difference between measurable exposure and meaningful exposure. While the metal concentrations measured in this study are not exceptionally high, we believe this study supports prior work identifying Mn and Ni exposure to be related to oil drilling in Los Angeles.

Human biomonitoring data shows the “toxic trespass” of harmful substances into the body, involuntarily violating bodies and homes and often disproportionally hurting low-income people of color.^76^ Although many polluters emit various harmful exposure and biomonitoring data indicates that many exposures are correlated, most environmental health studies typically focus on one exposure at a time. Mixture methods, like NMF, examine how exposures group together and the association between exposure mixtures and health outcomes. In this study, we identified industrial, oil drilling, and dietary source factors from the toenails of residents living near an urban oil drill site. Future community-based research studies should seek to advance methods that identify exposure sources to better understand how various environmental and industrial pollutants affect nearby residents.

## Supporting Information Available

Characteristics of participants by levels of each individual metal, metal concentrations by age, nickel concentrations by sex, map of study area, sensitivity analyses, and comparison of study results to another recent and nearby study (PDF)

## Abbreviations

NMF: non-negative matrix factorization
CA: California
As: arsenic
Cd: cadmium
Hg: mercury
Mn: manganese
Ni: nickel
Pb: lead
Sb: antimony

## References

[ref1] Bergbäck B.; Johansson K.; Mohlander U. Urban Metal Flows—A Case Study of Stockholm. Review and Conclusions. Water, Air, Soil Pollut.: Focus 2001, 1, 3–24. 10.1023/A:1017531532576.

[ref2] Luo X. S.; Yu S.; Zhu Y. G.; Li X. D. Trace metal contamination in urban soils of China. Sci. Total Environ. 2012, 421–422, 17–30. 10.1016/j.scitotenv.2011.04.020. 21575982

[ref3] Sadd J. L.; Shamasunder B. Oil Extraction in Los Angeles: Health, Land Use, and Environmental Justice Consequences. In Drilling Down: The Community Consequences of Expanded Oil Development in Los Angeles; Liberty Hill Foundation: Los Angeles, CA, USA, 2015.

[ref4] Garcia-Gonzales D. A.; Shonkoff S. B. C.; Hays J.; Jerrett M. Hazardous Air Pollutants Associated with Upstream Oil and Natural Gas Development: A Critical Synthesis of Current Peer-Reviewed Literature. Annu. Rev. Public Health 2019, 40, 283–304. 10.1146/annurev-publhealth-040218-043715. 30935307

[ref5] Johnston J. E.; Lim E.; Roh H. Impact of upstream oil extraction and environmental public health: A review of the evidence. Sci. Total Environ. 2019, 657, 187–199. 10.1016/j.scitotenv.2018.11.483. 30537580 PMC6344296

[ref6] Caron-Beaudoin É.; Bouchard M.; Wendling G.; et al. Urinary and hair concentrations of trace metals in pregnant women from Northeastern British Columbia, Canada: a pilot study. J. Exposure Sci. Environ. Epidemiol. 2019, 29, 613–623. 10.1038/s41370-019-0144-3. 31089244

[ref7] Lord C. J. Determination of trace metals in crude oil by inductively coupled plasma mass spectrometry with microemulsion sample introduction. Anal. Chem. 1991, 63, 1594–1599. 10.1021/ac00015a018.

[ref8] Ajayi T. R.; Torto N.; Tchokossa P.; Akinlua A. Natural radioactivity and trace metals in crude oils: implication for health. Environ. Geochem. Health 2009, 31, 61–69. 10.1007/s10653-008-9155-z. 18320332

[ref9] Gonzalez Alonso S.; Esteban-Hernandez J.; Valcarcel Rivera Y.; Hernandez-Barrera V.; Gil de Miguel A. [Water pollution in sources close to oil-producing fields of Bolivia]. Rev. Panam. Salud Publica 2010, 28, 235–243. 10.1590/s1020-49892010001000001. 21152710

[ref10] Fu X.; Cui Z.; Zang G. Migration, speciation and distribution of heavy metals in an oil-polluted soil affected by crude oil extraction processes. Environ. Sci.: Processes Impacts 2014, 16, 1737–44. 10.1039/c3em00618b. 24824116

[ref11] Asia I.; Jegede S.; Jegede D.; Ize-Iyamu O.; Akpasubi E. The effects of petroleum exploration and production operations on the heavy metals contents of soil and groundwater in the Niger Delta. Int. J. Phys. Sci. 2007, 2, 271–275.

[ref12] Johnston J. E.; Lim E.; Roh H. Impact of upstream oil extraction and environmental public health: A review of the evidence. Sci. Total Environ. 2019, 657, 187–199. 10.1016/j.scitotenv.2018.11.483. 30537580 PMC6344296

[ref13] McCarthy M. C.; Brown S. G.; Bai S., Baldwin Hills Air Quality Study Final Report; Sonoma Technology, Inc., 2015.

[ref14] OEHHA. CalEnviroScreen 3.0, 2018. https://oehha.ca.gov/calenviroscreen/report/calenviroscreen-30.

[ref15] Johnston J. E.; Enebish T.; Eckel S. P.; Navarro S.; Shamasunder B. Respiratory health, pulmonary function and local engagement in urban communities near oil development. Environ. Res. 2021, 197, 111088 10.1016/j.envres.2021.111088. 33794173 PMC8579779

[ref16] Gutiérrez-González E.; Garcia-Esquinas E.; de Larrea-Baz N. F.; et al. Toenails as biomarker of exposure to essential trace metals: A review. Environ. Res. 2019, 179, 108787 10.1016/j.envres.2019.108787. 31610392 PMC8164381

[ref17] Davis M. A.; Li Z.; Gilbert-Diamond D.; et al. Infant toenails as a biomarker of in utero arsenic exposure. J. Exposure Sci. Environ. Epidemiol. 2014, 24, 467–473. 10.1038/jes.2014.38. PMC4141012 24896769

[ref18] Signes-Pastor A. J.; Bouchard M. F.; Baker E.; Jackson B. P.; Karagas M. R. Toenail manganese as biomarker of drinking water exposure: a reliability study from a US pregnancy cohort. J. Exposure Sci. Environ. Epidemiol. 2019, 29, 648–654. 10.1038/s41370-018-0108-z. PMC6581634 30563963

[ref19] Signes-Pastor A. J.; Doherty B. T.; Romano M. E.; et al. Prenatal exposure to metal mixture and sex-specific birth outcomes in the New Hampshire Birth Cohort Study. Environ. Epidemiol. 2019, 3, e068 10.1097/EE9.0000000000000068. 31844832 PMC6914313

[ref20] El-Komy M.; Hafez V.; Hay R. A.; Mehaney D.; Hafez I. Toenail concentrations of zinc, selenium and nickel in patients with chronic recurrent warts: A pilot two-group comparative study. Indian J. Dermatol., Venereol. Leprol. 2019, 85, 51–55. 10.4103/ijdvl.IJDVL_861_17. 30226475

[ref21] Van Horne Y. O.; Farzan S. F.; Johnston J. E. Metal-mixtures in toenails of children living near an active industrial facility in Los Angeles County, California. J. Exposure Sci. Environ. Epidemiol. 2021, 31, 427–441. 10.1038/s41370-021-00330-8. PMC8893014 33935287

[ref22] Gaujoux R.; Seoighe C. A flexible R package for nonnegative matrix factorization. BMC Bioinf. 2010, 11, 367 10.1186/1471-2105-11-367. PMC2912887 20598126

[ref23] Diawara M. M.; Litt J. S.; Unis D.; et al. Arsenic, cadmium, lead, and mercury in surface soils, Pueblo, Colorado: implications for population health risk. Environ. Geochem. Health 2006, 28, 297–315. 10.1007/s10653-005-9000-6. 16752202

[ref24] Jones D. H.; Yu X.; Guo Q.; Duan X.; Jia C. Racial Disparities in the Heavy Metal Contamination of Urban Soil in the Southeastern United States. Int. J. Environ. Res. Public Health 2022, 19, 1105 10.3390/ijerph19031105. 35162130 PMC8834334

[ref25] Mielke H. W.; Laidlaw M. A.; Gonzales C. R. Estimation of leaded (Pb) gasoline’s continuing material and health impacts on 90 US urbanized areas. Environ. Int. 2011, 37, 248–257. 10.1016/j.envint.2010.08.006. 20825992

[ref26] Jones M. R.; Tellez-Plaza M.; Vaidya D.; et al. Ethnic, geographic and dietary differences in arsenic exposure in the multi-ethnic study of atherosclerosis (MESA. J. Exposure Sci. Environ. Epidemiol. 2019, 29, 310–322. 10.1038/s41370-018-0042-0. PMC6252166 29795237

[ref27] Cubadda F.; Jackson B. P.; Cottingham K. L.; Van Horne Y. O.; Kurzius-Spencer M. Human exposure to dietary inorganic arsenic and other arsenic species: State of knowledge, gaps and uncertainties. Sci. Total Environ. 2017, 579, 1228–1239. 10.1016/j.scitotenv.2016.11.108. 27914647 PMC5207036

[ref28] Rodríguez-Flores M.; Rodríguez-Castellón E. Lead and cadmium levels in soil and plants near highways and their correlation with traffic density. Environ. Pollut., Ser. B 1982, 4, 281–290. 10.1016/0143-148X(82)90014-3.

[ref29] Szwalec A.; Mundala P.; Kedzior R.; Pawlik J. Monitoring and assessment of cadmium, lead, zinc and copper concentrations in arable roadside soils in terms of different traffic conditions. Environ. Monit. Assess. 2020, 192, 155 10.1007/s10661-020-8120-x. 32006114 PMC6994438

[ref30] Dousova B.; Lhotka M.; Buzek F.; et al. Environmental interaction of antimony and arsenic near busy traffic nodes. Sci. Total Environ. 2020, 702, 134642 10.1016/j.scitotenv.2019.134642. 31734606

[ref31] Hogervorst J.; Plusquin M.; Vangronsveld J.; et al. House dust as possible route of environmental exposure to cadmium and lead in the adult general population. Environ. Res. 2007, 103, 30–37. 10.1016/j.envres.2006.05.009. 16843453

[ref32] Liu B.; Huang F.; Yu Y.; et al. Heavy Metals in Indoor Dust Across China: Occurrence, Sources and Health Risk Assessment. Arch. Environ. Contam. Toxicol. 2021, 81, 67–76. 10.1007/s00244-021-00849-9. 33944965

[ref33] Aelion C. M.; Davis H. T.; Lawson A. B.; Cai B.; McDermott S. Associations between soil lead concentrations and populations by race/ethnicity and income-to-poverty ratio in urban and rural areas. Environ. Geochem. Health 2013, 35, 1–12. 10.1007/s10653-012-9472-0. 22752852 PMC4655433

[ref34] Signes-Pastor A. J.; Gutierrez-Gonzalez E.; Garcia-Villarino M.; et al. Toenails as a biomarker of exposure to arsenic: A review. Environ. Res. 2021, 195, 110286 10.1016/j.envres.2020.110286. 33075355 PMC7987585

[ref35] Hayashi M.; Yamamoto K.; Yoshimura M.; Hayashi H.; Shitara A. Cadmium, lead, and zinc concentrations in human fingernails. Bull. Environ. Contam. Toxicol. 1993, 50, 547–553. 10.1007/BF00191244. 8467140

[ref36] Barker C. Chapter 2 Origin, Composition and Properties of Petroleum. In Developments in Petroleum Science, Donaldson E. C.; Chilingarian G. V.; Yen T. F., Eds.; Elsevier, 1985; pp 11–45.

[ref37] Filby R. H.; Shah A. K. The Nature of Metals in Petroleum. Neutron Activation Methods for Tace Elements in Crude Oils, Yen T. F., Ed.; Ann Arbor Science Publishers: Ann Arbor, 1975; pp 89–110.

[ref38] Curiale J. A. Distribution and Occurrence of Metals in Heavy Crude Oils and Solid Bitumens—Implications for Petroleum Exploration: Section II. Characterization, Maturation, and Degradation. Exploration for Heavy Crude Oil and Natural Bitumen: Studies in Geology; Datapages, Inc, 1987; pp 207–219.

[ref39] Drinking Water Quality Report. Los Angeles Department of Water & Power, 2018. https://www.csun.edu/sites/default/files/LADWP_Drinking_Water%20Quality_%20Report_2018.

[ref40] Martins A. C.; Krum B. N.; Queirós L.; et al. Manganese in the Diet: Bioaccessibility, Adequate Intake, and Neurotoxicological Effects. J. Agric. Food Chem. 2020, 68, 12893–12903. 10.1021/acs.jafc.0c00641. 32298096

[ref41] Genchi G.; Carocci A.; Lauria G.; Sinicropi M. S.; Catalano A. Nickel: Human Health and Environmental Toxicology. Int. J. Environ. Res. Public Health 2020, 17, 679 10.3390/ijerph17030679. PMC7037090 31973020

[ref42] Okoro E. E.; Ochonma C.; Omeje M.; et al. Radiological and toxicity risk exposures of oil based mud: health implication on drilling crew in Niger Delta. Environ. Sci. Pollut. Res. Int. 2020, 27, 5387–5397. 10.1007/s11356-019-07222-3. 31848949

[ref43] O’Callaghan-Gordo C.; Flores J. A.; Lizarraga P.; et al. Oil extraction in the Amazon basin and exposure to metals in indigenous populations. Environ. Res. 2018, 162, 226–230. 10.1016/j.envres.2018.01.013. 29407757

[ref44] Gonzalez D. J. X.; Francis C. K.; Shaw G. M.; Cullen M. R.; Baiocchi M.; Burke M. Upstream oil and gas production and ambient air pollution in California. Sci. Total Environ. 2022, 806, 150298 10.1016/j.scitotenv.2021.150298. 34844318

[ref45] Willis M. D.; Hill E. L.; Boslett A.; Kile M. L.; Carozza S. E.; Hystad P. Associations between Residential Proximity to Oil and Gas Drilling and Term Birth Weight and Small-for-Gestational-Age Infants in Texas: A Difference-in-Differences Analysis. Environ. Health Perspect. 2021, 129, 077002 10.1289/EHP7678. PMC8293911 34287013

[ref46] Elliott E. G.; Ma X.; Leaderer B. P.; et al. A community-based evaluation of proximity to unconventional oil and gas wells, drinking water contaminants, and health symptoms in Ohio. Environ. Res. 2018, 167, 550–557. 10.1016/j.envres.2018.08.022. 30145431

[ref47] Rabinowitz P. M.; Slizovskiy I. B.; Lamers V.; et al. Proximity to Natural Gas Wells and Reported Health Status: Results of a Household Survey in Washington County, Pennsylvania. Environ. Health Perspect. 2015, 123, 21–26. 10.1289/ehp.1307732. 25204871 PMC4286272

[ref48] Clark C. J.; Johnson N. P.; Soriano M.; et al. Unconventional Oil and Gas Development Exposure and Risk of Childhood Acute Lymphoblastic Leukemia: A Case–Control Study in Pennsylvania, 2009–2017. Environ. Health Perspect. 2022, 130, 087001 10.1289/EHP11092. PMC9383266 35975995

[ref49] Johnston J. E.; Okorn K.; Van Horne Y. O.; Jimenez A. Changes in neighborhood air quality after idling of an urban oil production site. Environ. Sci.: Processes Impacts 2021, 23, 967–980. 10.1039/d1em00048a. PMC12912187 34037015

[ref50] Tran K. V.; Casey J. A.; Cushing L. J.; Morello-Frosch R. Residential proximity to hydraulically fractured oil and gas wells and adverse birth outcomes in urban and rural communities in California (2006-2015). Environ. Epidemiol. 2021, 5, e172 10.1097/EE9.0000000000000172. 34909552 PMC8663888

[ref51] Zota A. R.; Schaider L. A.; Ettinger A. S.; Wright R. O.; Shine J. P.; Spengler J. D. Metal sources and exposures in the homes of young children living near a mining-impacted Superfund site. J. Exposure Sci. Environ. Epidemiol. 2011, 21, 495–505. 10.1038/jes.2011.21. PMC3161168 21587306

[ref52] Webb J.; Coomes O. T.; Ross N.; Mergler D. Mercury concentrations in urine of amerindian populations near oil fields in the peruvian and ecuadorian amazon. Environ. Res. 2016, 151, 344–350. 10.1016/j.envres.2016.07.040. 27525667

[ref53] Dahlgren J.; Takhar H.; Anderson-Mahoney P.; Kotlerman J.; Tarr J.; Warshaw R. Cluster of systemic lupus erythematosus (SLE) associated with an oil field waste site: a cross sectional study. Environ. Health 2007, 6, 8 10.1186/1476-069X-6-8. 17316448 PMC1821321

[ref54] Miedico O.; Iammarino M.; Paglia G.; Tarallo M.; Mangiacotti M.; Chiaravalle A. E. Environmental monitoring of the area surrounding oil wells in Val d’Agri (Italy): element accumulation in bovine and ovine organs. Environ. Monit. Assess. 2016, 188, 338 10.1007/s10661-016-5317-0. 27165602

[ref55] Pérez-Cadahía B.; Méndez J.; Pásaro E.; Lafuente A.; Cabaleiro T.; Laffon B. Biomonitoring of human exposure to prestige oil: effects on DNA and endocrine parameters. Environ. Health Insights 2008, 2, EHI-S954 10.4137/EHI.S954. PMC3091333 21572833

[ref56] Pérez-Cadahía B.; Laffon B.; Porta M.; et al. Relationship between blood concentrations of heavy metals and cytogenetic and endocrine parameters among subjects involved in cleaning coastal areas affected by the ‘Prestige’tanker oil spill. Chemosphere 2008, 71, 447–455. 10.1016/j.chemosphere.2007.10.053. 18221981

[ref57] Lee S. G.; Kang I.; Seo M. N.; et al. Exposure Levels and Contributing Factors of Various Arsenic Species and Their Health Effects on Korean Adults. Arch. Environ. Contam. Toxicol. 2022, 82, 391–402. 10.1007/s00244-022-00913-y. 35132447

[ref58] Pérez R.; Domenech E.; Conchado A.; Sanchez A.; Coscolla C.; Yusa V. Influence of diet in urinary levels of metals in a biomonitoring study of a child population of the Valencian region (Spain). Sci. Total Environ. 2018, 618, 1647–1657. 10.1016/j.scitotenv.2017.10.011. 29054627

[ref59] Salcedo-Bellido I.; Gutierrez-Gonzalez E.; Garcia-Esquinas E.; et al. Toxic metals in toenails as biomarkers of exposure: A review. Environ. Res. 2021, 197, 111028 10.1016/j.envres.2021.111028. 33753073

[ref60] Terry A. L.; Herrick K. A.; Afful J.; Ahluwalia N. Seafood Consumption in the United States, 2013-2016; US Department of Health & Human Services, Centers for Disease Control and Prevention, National Center for Health Statistics, 2018; Vol. 321, https://www.ncbi.nlm.nih.gov/pubmed/30312150.

[ref61] Baldantoni D.; Morra L.; Zaccardelli M.; Alfani A. Cadmium accumulation in leaves of leafy vegetables. Ecotoxicol. Environ. Saf. 2016, 123, 89–94. 10.1016/j.ecoenv.2015.05.019. 26004982

[ref62] Adams S. V.; Newcomb P. A.; Shafer M. M.; et al. Sources of cadmium exposure among healthy premenopausal women. Sci. Total Environ. 2011, 409, 1632–1637. 10.1016/j.scitotenv.2011.01.037. 21333327 PMC3056571

[ref63] Fløtre C. H.; Varsi K.; Helm T.; Bolann B.; Bjorke-Monsen A. L. Predictors of mercury, lead, cadmium and antimony status in Norwegian never-pregnant women of fertile age. PLoS One 2017, 12, e0189169 10.1371/journal.pone.0189169. 29206878 PMC5716542

[ref64] Sanders A. P.; Miller S. K.; Nguyen V.; Kotch J. B.; Fry R. C. Toxic metal levels in children residing in a smelting craft village in Vietnam: a pilot biomonitoring study. BMC Public Health 2014, 14, 114 10.1186/1471-2458-14-114. 24495283 PMC3922956

[ref65] Vorvolakos T.; Arseniou S.; Samakouri M. There is no safe threshold for lead exposure: Alpha literature review. Psychiatriki 2016, 27, 204–214. 10.22365/jpsych.2016.273.204. 27837574

[ref66] Rehman K.; Fatima F.; Waheed I.; Akash M. S. H. Prevalence of exposure of heavy metals and their impact on health consequences. J. Cell. Biochem. 2018, 119, 157–184. 10.1002/jcb.26234. 28643849

[ref67] Andrew A. S.; Mason R. A.; Memoli V.; Duell E. J. Arsenic activates EGFR pathway signaling in the lung. Toxicol. Sci. 2009, 109, 350–357. 10.1093/toxsci/kfp015. 19168569 PMC2683921

[ref68] Farzan S. F.; Chen Y.; Rees J. R.; Zens M. S.; Karagas M. R. Risk of death from cardiovascular disease associated with low-level arsenic exposure among long-term smokers in a US population-based study. Toxicol. Appl. Pharmacol. 2015, 287, 93–97. 10.1016/j.taap.2015.05.013. 26048586 PMC4536141

[ref69] Farzan S. F.; Li Z.; Korrick S. A.; et al. Infant Infections and Respiratory Symptoms in Relation to in Utero Arsenic Exposure in a U.S. Cohort. Environ. Health Perspect. 2016, 124, 840–847. 10.1289/ehp.1409282. 26359651 PMC4892909

[ref70] Farzan S. F.; Gossai A.; Chen Y.; Chasan-Taber L.; Baker E.; Karagas M. Maternal arsenic exposure and gestational diabetes and glucose intolerance in the New Hampshire birth cohort study. Environ. Health 2016, 15, 106 10.1186/s12940-016-0194-0. 27825389 PMC5101688

[ref71] Levy B. S.; Nassetta W. J. Neurologic effects of manganese in humans: a review. Int. J. Occup. Environ. Health 2003, 9, 153–163. 10.1179/oeh.2003.9.2.153. 12848244

[ref72] Hansen A. F.; Simić A.; Åsvold B. O.; et al. Trace elements in early phase type 2 diabetes mellitus-A population-based study. The HUNT study in Norway. J. Trace Elem. Med. Biol. 2017, 40, 46–53. 10.1016/j.jtemb.2016.12.008. 28159221

[ref73] Liu G.; Sun L.; Pan A.; et al. Nickel exposure is associated with the prevalence of type 2 diabetes in Chinese adults. Int. J. Epidemiol. 2015, 44, 240–248. 10.1093/ije/dyu200. 25324152

[ref74] Zierold K. M.; Myers J. V.; Brock G. N.; Sears C. G.; Sears L. L.; Zhang C. H. Nail Samples of Children Living near Coal Ash Storage Facilities Suggest Fly Ash Exposure and Elevated Concentrations of Metal(loid)s. Environ. Sci. Technol. 2021, 55, 9074–9086. 10.1021/acs.est.1c01541. 34132542 PMC10725724

[ref75] Madrigal J. M.; Persky V.; Jackson B. P.; et al. Assessment of Metal Concentrations and Associations with Pulmonary Function among Children with Asthma in Chicago, Illinois. Int. J. Environ. Res. Public Health 2021, 18, 7279 10.3390/ijerph18147279. 34299734 PMC8307469

[ref76] Brown P.; De La Rose V.; Cordner A. Toxic Trespass: Science, Activism, and Policy Concerning Chemicals in Our Bodies. In Toxic Truths: Environmental Justice and Citizen Science in a Post-Truth Age, Davies T.; Mah A., Eds.; Manchester University Press: Manchester, 2020; pp 34–58.

