# Supplementary material for: Metal Exposures in Residents Living Near an Urban Oil Drilling Site in Los Angeles, California
Source: Environ Sci Technol. 2022 Oct 26;56(22):15981–9. doi: 10.1021/acs.est.2c04926 (PMC9670842; doi:10.1021/acs.est.2c04926)
Supplement: Supplementary file 1 — es2c04926_si_001.pdf [file es2c04926_si_001.pdf]

Supporting Information

Metal exposures in residents living near an urban oil drilling site in Los Angeles, California

Arbor J.L. Quist, Yoshira Ornelas Van Horne, Shohreh F. Farzan, Jill E. Johnston

Summary of Supporting Information– 15 pages

Figure 1. Ni concentration by sex
Figure 2. NMF results when restricted to adults
Figure 3. NMF results when restricted to participants under 18
Figure 4. NMF results when analyses accounted for participants who live in the same household
Figure 6. Map of oil drill site, nearby highway, and participant homes
Figure 7. Metal concentrations in toenails and age

Table 1. Characteristics of participants by low, medium, and high As concentration
Table 2. Characteristics of participants by low, medium, and high Cd concentration
Table 3. Characteristics of participants by low, medium, and high Hg concentration
Table 4. Characteristics of participants by low, medium, and high Mn concentration
Table 5. Characteristics of participants by low, medium, and high Ni concentration
Table 6. Characteristics of participants by low, medium, and high Pb concentration
Table 7. Characteristics of participants by low, medium, and high Sb concentration
Table 8. Comparing metal concentrations of children in this study to Van Horne et al. 2021

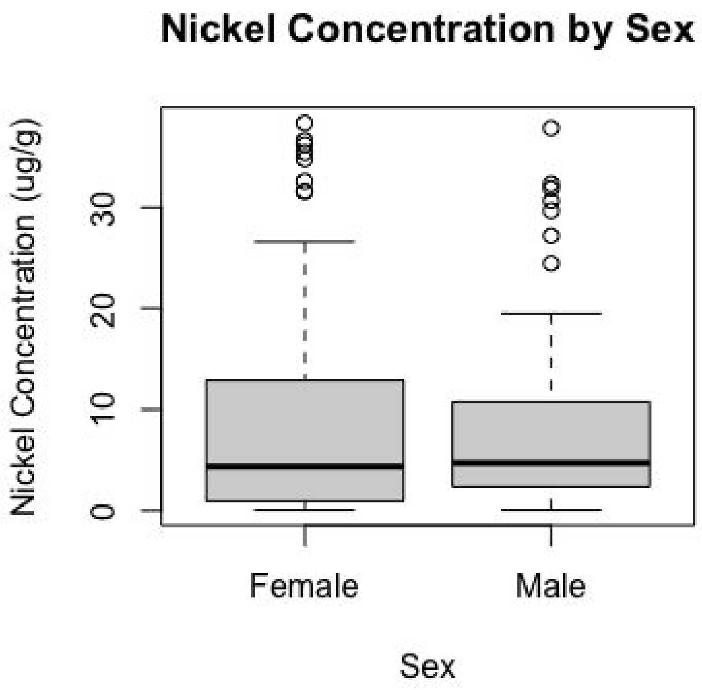

Supplementary Figure 1. Ni concentration by sex (n=203). While some studies have observed Ni differences by sex, we observed similar concentration distributions. (Of the 20 participants excluded for high Ni concentrations, 12 were male and 7 were female).

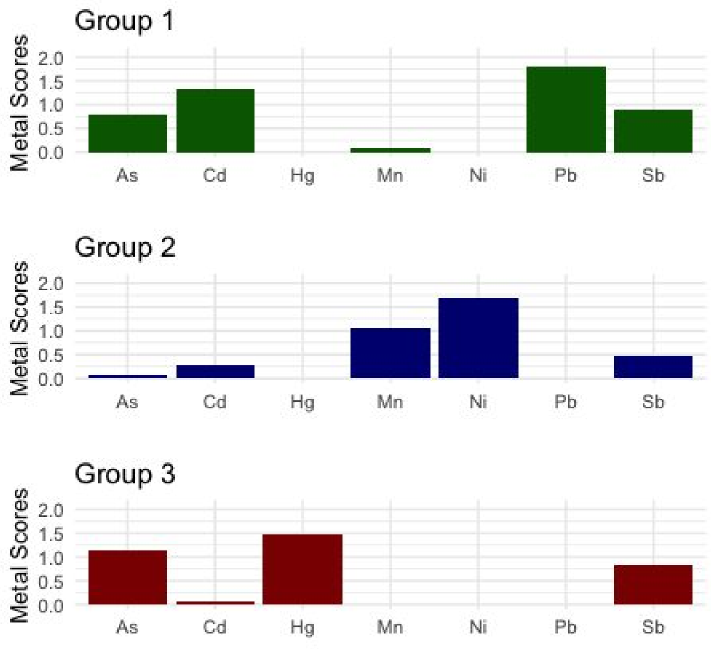

Supplementary Figure 2. NMF results when restricted to adults (n=162)

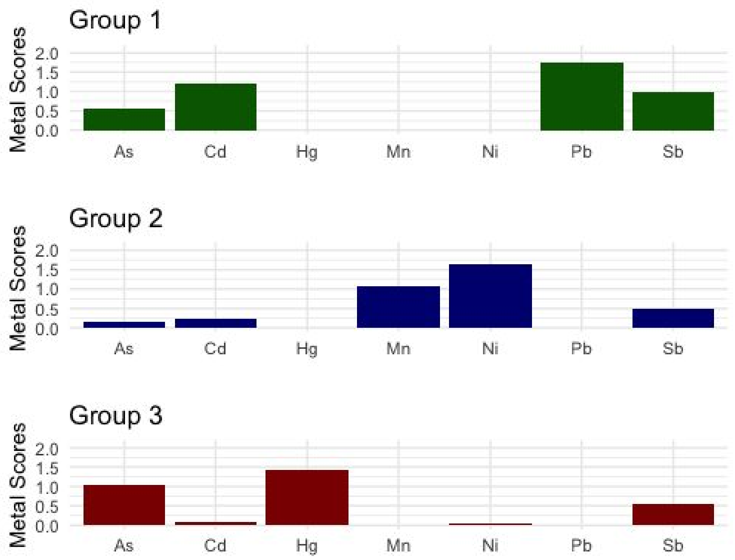

Supplementary Figure 3. NMF results when restricted to participants under 18 (n=41).

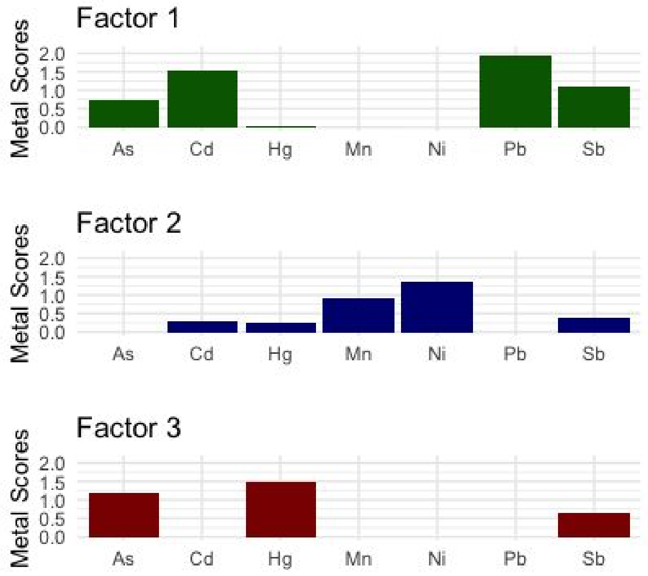

Supplementary Figure 4. NMF results when analyses accounted for participants who live in the same household. In our models that adjusted for participant age, we added a fixed effect for house ID (using lme4 in R), and then used these residuals as adjusted metal concentrations in NMF. These results are similar to our main results.

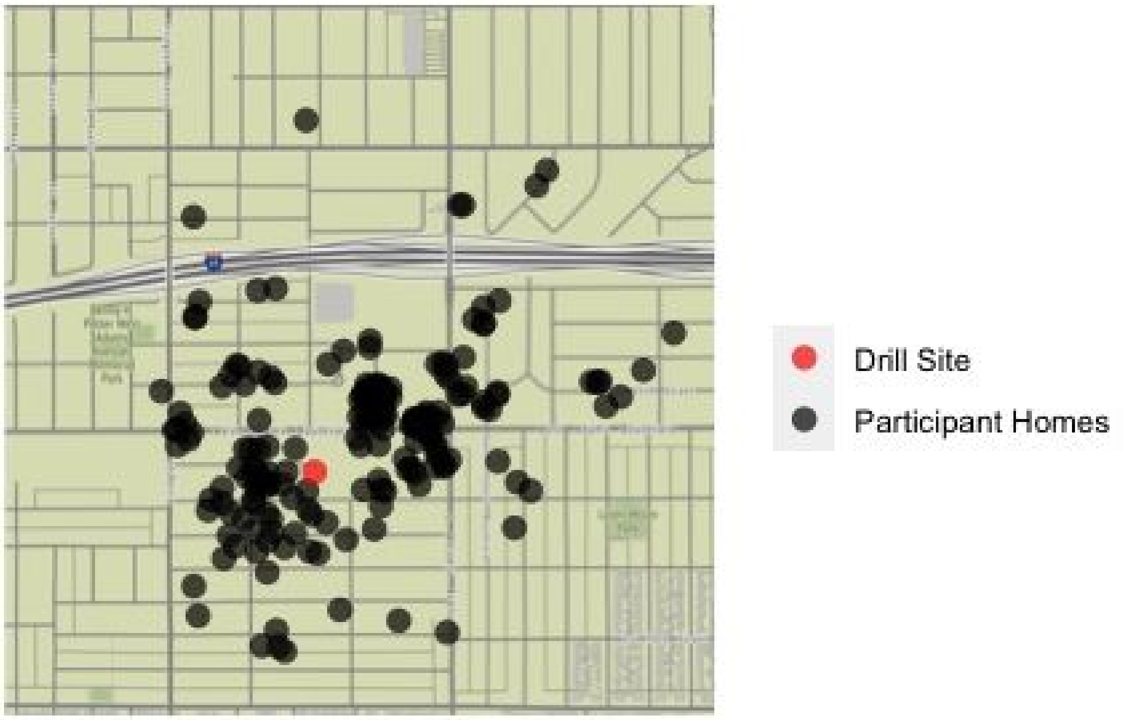

Supplementary Figure 5. Map of oil drill site, nearby highway, and participant homes. Participant addresses have been randomly jittered using position_jitter() in R to maintain participant confidentiality.

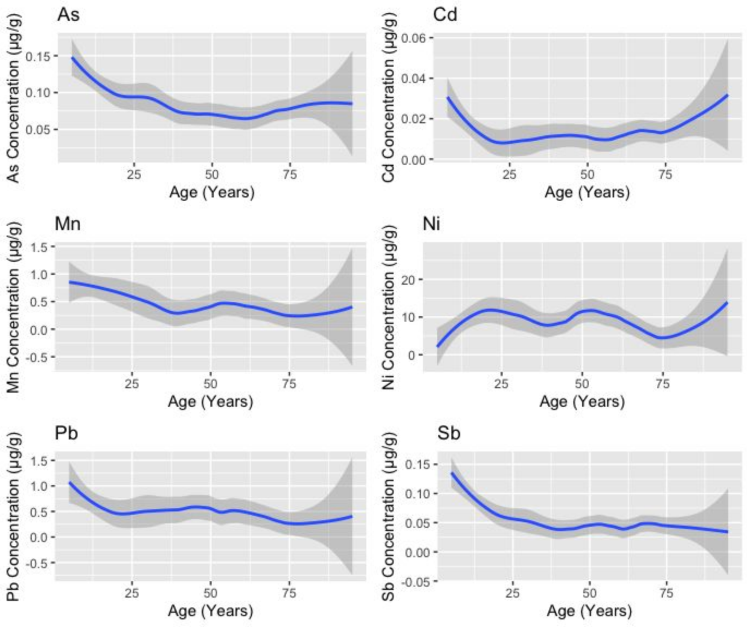

Supplementary Figure 6. Metal concentrations in toenails and age.

Supplementary Table 1. Characteristics of participants by low, medium, and high As concentration.

**As**		Low	Medium	High	
	level	(0.0114,0.0573]	(0.0573,0.0908]	(0.0908,0.339]	p
N		66	67	67	
Age	≤10	2 (3.0)	2 (3.0)	14 (20.9)	0.003
	10-18	6 (9.1)	5 (7.5)	12 (17.9)	
	19-30	3 (4.5)	6 (9.0)	4 (6.0)	
	31-40	7 (10.6)	6 (9.0)	4 (6.0)	
	41-50	11 (16.7)	6 (9.0)	7 (10.4)	
	51-60	15 (22.7)	11 (16.4)	6 (9.0)	
	>60	22 (33.3)	31 (46.3)	20 (29.9)	
Sex	Female	45 (68.2)	37 (55.2)	41 (61.2)	0.307
	Male	21 (31.8)	30 (44.8)	26 (38.8)	
Race/ethnicity	Asian	1 (1.5)	7 (10.4)	10 (14.9)	0.032
	Black or African American	18 (27.3)	18 (26.9)	7 (10.4)	
	Hispanic or Latino	43 (65.2)	38 (56.7)	47 (70.1)	
	Other/Multi-racial	4 (6.1)	4 (6.0)	3 (4.5)	
Recent smoker	No	62 (93.9)	60 (89.6)	65 (97.0)	0.212
	Yes	4 (6.1)	7 (10.4)	2 (3.0)	
Ever cigarettes	No	44 (66.7)	38 (57.6)	56 (83.6)	0.004
	Yes	22 (33.3)	28 (42.4)	11 (16.4)	
Distance to Well		359.69 (199.16)	329.09 (195.41)	379.43 (241.41)	0.389
Distance to Well	200-1000m	54 (81.8)	42 (62.7)	49 (73.1)	0.047
	<200m	12 (18.2)	25 (37.3)	18 (26.9)	
Direction of well	east	39 (59.1)	40 (59.7)	41 (61.2)	0.968
	west	27 (40.9)	27 (40.3)	26 (38.8)	
Distance/direction well	downwind, <200m	10 (15.2)	21 (31.3)	15 (22.4)	0.333
	downwind, 200-1000m	29 (43.9)	19 (28.4)	26 (38.8)	

	upwind, <200m	2 (3.0)	4 (6.0)	3 (4.5)	
	upwind, 200-1000m	25 (37.9)	23 (34.3)	23 (34.3)	
Distance of highway (mean)		548.19 (222.11)	501.39 (237.30)	457.12 (169.11)	0.048
Distance of highway	<500m	26 (39.4)	38 (56.7)	36 (53.7)	0.103
	≥500m	40 (60.6)	29 (43.3)	31 (46.3)	
Apartment	No	30 (45.5)	38 (56.7)	36 (53.7)	0.404
	Yes	36 (54.5)	29 (43.3)	31 (46.3)	
Above 3rd Floor	No	58 (87.9)	54 (80.6)	55 (82.1)	0.490
	Yes	8 (12.1)	13 (19.4)	12 (17.9)	

Supplementary Table 2. Characteristics of participants by low, medium, and high Cd concentration.

**Cd**		Low	Medium	High	
	level	(0.000637,0.00442]	(0.00442,0.0112]	(0.0112,0.11]	p
N		66	67	67	
Age	≤10	1 (1.5)	2 (3.0)	15 (22.4)	<0.001
	10-18	8 (12.1)	7 (10.4)	7 (10.4)	
	19-30	2 (3.0)	7 (10.4)	4 (6.0)	
	31-40	4 (6.1)	11 (16.4)	2 (3.0)	
	41-50	13 (19.7)	3 (4.5)	8 (11.9)	
	51-60	13 (19.7)	12 (17.9)	7 (10.4)	
	>60	25 (37.9)	25 (37.3)	24 (35.8)	
Sex	Female	37 (56.1)	45 (67.2)	42 (62.7)	0.415
	Male	29 (43.9)	22 (32.8)	25 (37.3)	
Race/ethnicity	Asian	5 (7.6)	8 (11.9)	5 (7.5)	0.775
	Black or African American	13 (19.7)	12 (17.9)	18 (26.9)	
	Hispanic or Latino	43 (65.2)	44 (65.7)	41 (61.2)	
	Other/Multi-racial	5 (7.6)	3 (4.5)	3 (4.5)	
Recent smoker	No	62 (93.9)	64 (95.5)	61 (91.0)	0.567
	Yes	4 (6.1)	3 (4.5)	6 (9.0)	

Ever cigarettes	No	40 (60.6)	50 (75.8)	48 (71.6)	0.149
	Yes	26 (39.4)	16 (24.2)	19 (28.4)	
Distance to Well		351.76 (224.42)	323.79 (175.67)	391.75 (232.83)	0.180
Distance to Well	200-1000m	48 (72.7)	45 (67.2)	51 (76.1)	0.507
	<200m	18 (27.3)	22 (32.8)	16 (23.9)	
Direction of well	east	36 (54.5)	41 (61.2)	43 (64.2)	0.510
	west	30 (45.5)	26 (38.8)	24 (35.8)	
Distance/direction well	downwind, <200m	16 (24.2)	16 (23.9)	15 (22.4)	0.284
	downwind, 200-1000m	20 (30.3)	25 (37.3)	28 (41.8)	
	upwind, <200m	2 (3.0)	6 (9.0)	1 (1.5)	
	upwind, 200-1000m	28 (42.4)	20 (29.9)	23 (34.3)	
Distance of highway (mean)		511.94 (197.03)	509.94 (203.24)	482.52 (240.33)	0.676
Distance of highway	<500m	32 (48.5)	32 (47.8)	37 (55.2)	0.636
	≥500m	34 (51.5)	35 (52.2)	30 (44.8)	
Apartment	No	32 (48.5)	40 (59.7)	32 (47.8)	0.301
	Yes	34 (51.5)	27 (40.3)	35 (52.2)	
Above 3rd Floor	No	57 (86.4)	56 (83.6)	54 (80.6)	0.669
	Yes	9 (13.6)	11 (16.4)	13 (19.4)	

Supplementary Table 3. Characteristics of participants by low, medium, and high Hg concentration.

**Hg**		Low	Medium	High	
	level	(0.00354,0.0323]	(0.0323,0.0786]	(0.0786,0.52]	p
N		53	67	67	
Age	≤10	4 (7.5)	8 (11.9)	6 (9.0)	0.601
	10-18	7 (13.2)	9 (13.4)	7 (10.4)	
	19-30	5 (9.4)	3 (4.5)	5 (7.5)	
	31-40	6 (11.3)	6 (9.0)	2 (3.0)	
	41-50	4 (7.5)	9 (13.4)	8 (11.9)	
	51-60	8 (15.1)	13 (19.4)	8 (11.9)	

	>60	19 (35.8)	19 (28.4)	31 (46.3)	
Sex	Female	34 (64.2)	41 (61.2)	35 (52.2)	0.372
	Male	19 (35.8)	26 (38.8)	32 (47.8)	
Race/ethnicity	Asian	1 (1.9)	1 (1.5)	16 (23.9)	<0.001
	Black or African American	6 (11.3)	17 (25.4)	18 (26.9)	
	Hispanic or Latino	44 (83.0)	46 (68.7)	27 (40.3)	
	Other/Multi-racial	2 (3.8)	3 (4.5)	6 (9.0)	
Recent smoker	No	49 (92.5)	62 (92.5)	63 (94.0)	0.925
	Yes	4 (7.5)	5 (7.5)	4 (6.0)	
Ever cigarettes	No	39 (73.6)	45 (68.2)	43 (64.2)	0.546
	Yes	14 (26.4)	21 (31.8)	24 (35.8)	
Distance to Well		396.37 (252.00)	370.60 (217.56)	321.41 (183.13)	0.154
Distance to Well	200-1000m	43 (81.1)	54 (80.6)	36 (53.7)	<0.001
	<200m	10 (18.9)	13 (19.4)	31 (46.3)	
Direction of well	east	31 (58.5)	43 (64.2)	42 (62.7)	0.808
	west	22 (41.5)	24 (35.8)	25 (37.3)	
Distance/direction well	downwind, <200m	8 (15.1)	11 (16.4)	26 (38.8)	0.009
	downwind, 200-1000m	23 (43.4)	32 (47.8)	16 (23.9)	
	upwind, <200m	2 (3.8)	2 (3.0)	5 (7.5)	
	upwind, 200-1000m	20 (37.7)	22 (32.8)	20 (29.9)	
Distance of highway (mean)		496.11 (229.83)	535.09 (220.71)	466.98 (192.85)	0.184
Distance of highway	<500m	25 (47.2)	27 (40.3)	44 (65.7)	0.010
	≥500m	28 (52.8)	40 (59.7)	23 (34.3)	
Apartment	No	29 (54.7)	37 (55.2)	32 (47.8)	0.636
	Yes	24 (45.3)	30 (44.8)	35 (52.2)	
Above 3rd Floor	No	46 (86.8)	61 (91.0)	48 (71.6)	0.008
	Yes	7 (13.2)	6 (9.0)	19 (28.4)	

Supplementary Table 4. Characteristics of participants by low, medium, and high Mn concentration.

**Mn**		Low	Medium	High	
	level	(0.0239,0.189]	(0.189,0.407]	(0.407,8.24]	p
N		66	67	67	
Age	≤10	1 (1.5)	0 (0.0)	17 (25.4)	<0.001
	10-18	1 (1.5)	10 (14.9)	12 (17.9)	
	19-30	3 (4.5)	8 (11.9)	2 (3.0)	
	31-40	3 (4.5)	10 (14.9)	4 (6.0)	
	41-50	11 (16.7)	7 (10.4)	5 (7.5)	
	51-60	12 (18.2)	9 (13.4)	11 (16.4)	
	>60	35 (53.0)	23 (34.3)	16 (23.9)	
Sex	Female	44 (66.7)	43 (64.2)	36 (53.7)	0.265
	Male	22 (33.3)	24 (35.8)	31 (46.3)	
Race/ethnicity	Asian	10 (15.2)	6 (9.0)	2 (3.0)	0.047
	Black or African American	16 (24.2)	18 (26.9)	9 (13.4)	
	Hispanic or Latino	38 (57.6)	38 (56.7)	52 (77.6)	
	Other/Multi-racial	2 (3.0)	5 (7.5)	4 (6.0)	
Recent smoker	No	62 (93.9)	62 (92.5)	63 (94.0)	0.926
	Yes	4 (6.1)	5 (7.5)	4 (6.0)	
Ever cigarettes	No	37 (56.9)	45 (67.2)	56 (83.6)	0.004
	Yes	28 (43.1)	22 (32.8)	11 (16.4)	
Distance to Well		314.73 (183.33)	355.21 (213.28)	396.89 (234.59)	0.084
Distance to Well	200-1000m	42 (63.6)	46 (68.7)	56 (83.6)	0.028
	<200m	24 (36.4)	21 (31.3)	11 (16.4)	
Direction of well	east	41 (62.1)	41 (61.2)	39 (58.2)	0.890
	west	25 (37.9)	26 (38.8)	28 (41.8)	
Distance/direction well	downwind, <200m	22 (33.3)	16 (23.9)	9 (13.4)	0.108
	downwind, 200-1000m	19 (28.8)	25 (37.3)	30 (44.8)	

	upwind, <200m	2 (3.0)	5 (7.5)	2 (3.0)	
	upwind, 200-1000m	23 (34.8)	21 (31.3)	26 (38.8)	
Distance of highway (mean)		485.59 (183.32)	519.41 (230.08)	496.98 (225.56)	0.651
Distance of highway	<500m	37 (56.1)	35 (52.2)	29 (43.3)	0.318
	≥500m	29 (43.9)	32 (47.8)	38 (56.7)	
Apartment	No	26 (39.4)	38 (56.7)	40 (59.7)	0.041
	Yes	40 (60.6)	29 (43.3)	27 (40.3)	
Above 3rd Floor	No	50 (75.8)	59 (88.1)	58 (86.6)	0.114
	Yes	16 (24.2)	8 (11.9)	9 (13.4)	

Supplementary Table 5. Characteristics of participants by low, medium, and high Ni concentration.

**Ni**		Low	Medium	High	
	level	(0.0545,2.33]	(2.33,8.88]	(8.88,38.4]	p
N		66	67	67	
Age	≤10	5 (7.6)	10 (14.9)	3 (4.5)	0.206
	10-18	7 (10.6)	7 (10.4)	9 (13.4)	
	19-30	2 (3.0)	6 (9.0)	5 (7.5)	
	31-40	9 (13.6)	2 (3.0)	6 (9.0)	
	41-50	5 (7.6)	8 (11.9)	10 (14.9)	
	51-60	8 (12.1)	11 (16.4)	13 (19.4)	
	>60	30 (45.5)	23 (34.3)	21 (31.3)	
Sex	Female	48 (72.7)	32 (47.8)	44 (65.7)	0.009
	Male	18 (27.3)	35 (52.2)	23 (34.3)	
Race/ethnicity	Asian	7 (10.6)	8 (11.9)	3 (4.5)	0.321
	Black or African American	11 (16.7)	16 (23.9)	16 (23.9)	
	Hispanic or Latino	46 (69.7)	41 (61.2)	42 (62.7)	
	Other/Multi-racial	2 (3.0)	2 (3.0)	6 (9.0)	
Recent smoker	No	64 (97.0)	61 (91.0)	62 (92.5)	0.354
	Yes	2 (3.0)	6 (9.0)	5 (7.5)	

Ever cigarettes	No	49 (74.2)	47 (70.1)	42 (63.6)	0.411
	Yes	17 (25.8)	20 (29.9)	24 (36.4)	
Distance to Well		305.18 (172.64)	368.43 (235.17)	393.89 (218.96)	0.046
Distance to Well	200-1000m	45 (68.2)	47 (70.1)	53 (79.1)	0.322
	<200m	21 (31.8)	20 (29.9)	14 (20.9)	
Direction of well	east	47 (71.2)	45 (67.2)	29 (43.3)	0.002
	west	19 (28.8)	22 (32.8)	38 (56.7)	
Distance/direction well	downwind, <200m	19 (28.8)	17 (25.4)	11 (16.4)	0.038
	downwind, 200-1000m	28 (42.4)	28 (41.8)	18 (26.9)	
	upwind, <200m	2 (3.0)	3 (4.5)	3 (4.5)	
	upwind, 200-1000m	17 (25.8)	19 (28.4)	35 (52.2)	
Distance of highway (mean)		487.11 (161.61)	474.68 (183.40)	538.59 (274.14)	0.184
Distance of highway	<500m	35 (53.0)	34 (50.7)	32 (47.8)	0.830
	≥500m	31 (47.0)	33 (49.3)	35 (52.2)	
Apartment	No	26 (39.4)	34 (50.7)	44 (65.7)	0.010
	Yes	40 (60.6)	33 (49.3)	23 (34.3)	
Above 3rd Floor	No	53 (80.3)	55 (82.1)	59 (88.1)	0.450
	Yes	13 (19.7)	12 (17.9)	8 (11.9)	

Supplementary Table 6. Characteristics of participants by low, medium, and high Pb concentration.

**Pb**		Low	Medium	High	
	level	(0.0157,0.146]	(0.146,0.4]	(0.4,4.59]	p
N		66	67	67	
Age	≤10	2 (3.0)	0 (0.0)	16 (23.9)	<0.001
	10-18	5 (7.6)	5 (7.5)	13 (19.4)	
	19-30	1 (1.5)	10 (14.9)	2 (3.0)	
	31-40	6 (9.1)	8 (11.9)	3 (4.5)	
	41-50	12 (18.2)	7 (10.4)	5 (7.5)	
	51-60	12 (18.2)	12 (17.9)	8 (11.9)	

	>60	28 (42.4)	25 (37.3)	20 (29.9)	
Sex	Female	40 (60.6)	45 (67.2)	38 (56.7)	0.454
	Male	26 (39.4)	22 (32.8)	29 (43.3)	
Race/ethnicity	Asian	7 (10.6)	9 (13.4)	2 (3.0)	0.424
	Black or African American	14 (21.2)	16 (23.9)	13 (19.4)	
	Hispanic or Latino	42 (63.6)	38 (56.7)	48 (71.6)	
	Other/Multi-racial	3 (4.5)	4 (6.0)	4 (6.0)	
Recent smoker	No	60 (90.9)	65 (97.0)	62 (92.5)	0.334
	Yes	6 (9.1)	2 (3.0)	5 (7.5)	
Ever cigarettes	No	38 (57.6)	48 (71.6)	52 (78.8)	0.027
	Yes	28 (42.4)	19 (28.4)	14 (21.2)	
Distance to Well		311.58 (172.83)	340.20 (212.70)	415.71 (237.33)	0.014
Distance to Well	200-1000m	46 (69.7)	41 (61.2)	58 (86.6)	0.004
	<200m	20 (30.3)	26 (38.8)	9 (13.4)	
Direction of well	east	42 (63.6)	40 (59.7)	38 (56.7)	0.716
	west	24 (36.4)	27 (40.3)	29 (43.3)	
Distance/direction well	downwind, <200m	19 (28.8)	18 (26.9)	9 (13.4)	0.003
	downwind, 200-1000m	23 (34.8)	22 (32.8)	29 (43.3)	
	upwind, <200m	1 (1.5)	8 (11.9)	0 (0.0)	
	upwind, 200-1000m	23 (34.8)	19 (28.4)	29 (43.3)	
Distance of highway (mean)		501.14 (183.84)	520.34 (225.29)	484.53 (230.17)	0.626
Distance of highway	<500m	32 (48.5)	36 (53.7)	32 (47.8)	0.753
	≥500m	34 (51.5)	31 (46.3)	35 (52.2)	
Apartment	No	26 (39.4)	37 (55.2)	41 (61.2)	0.034
	Yes	40 (60.6)	30 (44.8)	26 (38.8)	
Above 3rd Floor	No	52 (78.8)	58 (86.6)	57 (85.1)	0.440
	Yes	14 (21.2)	9 (13.4)	10 (14.9)	

Supplementary Table 7. Characteristics of participants by low, medium, and high Sb concentration.

**Sb**		Low	Medium	High	
	level	(0.00294,0.0247]	(0.0247,0.0541]	(0.0541,0.273]	p
N		66	67	67	
Age	≤10	1 (1.5)	2 (3.0)	15 (22.4)	<0.001
	10-18	3 (4.5)	5 (7.5)	15 (22.4)	
	19-30	3 (4.5)	4 (6.0)	6 (9.0)	
	31-40	8 (12.1)	6 (9.0)	3 (4.5)	
	41-50	13 (19.7)	9 (13.4)	2 (3.0)	
	51-60	10 (15.2)	14 (20.9)	8 (11.9)	
	>60	28 (42.4)	27 (40.3)	18 (26.9)	
Sex	Female	41 (62.1)	44 (65.7)	39 (58.2)	0.673
	Male	25 (37.9)	23 (34.3)	28 (41.8)	
Race/ethnicity	Asian	9 (13.6)	9 (13.4)	0 (0.0)	0.032
	Black or African American	12 (18.2)	15 (22.4)	16 (23.9)	
	Hispanic or Latino	39 (59.1)	42 (62.7)	47 (70.1)	
	Other/Multi-racial	6 (9.1)	1 (1.5)	4 (6.0)	
Recent smoker	No	63 (95.5)	62 (92.5)	62 (92.5)	0.734
	Yes	3 (4.5)	5 (7.5)	5 (7.5)	
Ever cigarettes	No	39 (60.0)	44 (65.7)	55 (82.1)	0.016
	Yes	26 (40.0)	23 (34.3)	12 (17.9)	
Distance to Well		326.94 (209.77)	348.12 (201.07)	392.65 (225.38)	0.192
Distance to Well	200-1000m	41 (62.1)	47 (70.1)	57 (85.1)	0.011
	<200m	25 (37.9)	20 (29.9)	10 (14.9)	
Direction of well	east	40 (60.6)	45 (67.2)	35 (52.2)	0.210
	west	26 (39.4)	22 (32.8)	32 (47.8)	
Distance/direction well	downwind, <200m	19 (28.8)	17 (25.4)	10 (14.9)	0.026
	downwind, 200-1000m	21 (31.8)	28 (41.8)	25 (37.3)	
	upwind, <200m	6 (9.1)	3 (4.5)	0 (0.0)	

	upwind, 200-1000m	20 (30.3)	19 (28.4)	32 (47.8)	
Distance of highway (mean)		533.47 (215.46)	494.32 (225.59)	478.70 (198.65)	0.316
Distance of highway	<500m	31 (47.0)	36 (53.7)	33 (49.3)	0.730
	≥500m	35 (53.0)	31 (46.3)	34 (50.7)	
Apartment	No	31 (47.0)	36 (53.7)	38 (56.7)	0.515
	Yes	35 (53.0)	31 (46.3)	29 (43.3)	
Above 3rd Floor	No	53 (80.3)	57 (85.1)	57 (85.1)	0.694
	Yes	13 (19.7)	10 (14.9)	10 (14.9)	

Supplementary Table 8. Comparing metal concentrations of children in this study to Van Horne et al. 2021.

Metal	Van Horne et al. 2021 Mean ± SD (µg/g)	Current study – children only Mean ± SD (µg/g)
As	0.23 ± 0.20	0.12 ± 0.07
Cd	0.05 ± 0.08	0.19 ± 0.02
Mn	1.72 ± 1.65	0.88 ± 1.34
Pb	0.84 ± 0.89	0.73 ± 0.61
Sb	0.18 ± 0.18	0.10 ± 0.06
